# A Feedback Active Control Approach to Road Noise Based on a Single Microphone Sensor to Improve Automotive Cabin Sound Comfort

**DOI:** 10.3390/s24082515

**Published:** 2024-04-14

**Authors:** Hao Liu, Jaecheon Lee

**Affiliations:** Department of Mechanical Engineering, Keimyung University, Daegu 42601, Republic of Korea; ljcds@kmu.ac.kr

**Keywords:** automotive cabin sound comfort, feedback active noise control (ANC), Filtered-x Least Mean Square (FxLMS), online secondary path modeling, single microphone sensor

## Abstract

Tire–road noise deteriorates the sound quality of a vehicle’s interior and affects the driving safety and comfort. Obtaining low interior noise is a challenge for passenger car manufacturers. Traditional passive noise control (PNC) is efficient for canceling high frequency noise but not useful for low frequency noise, while active noise control (ANC), according to the residual error signal, can generate an anti-noise signal to reduce the original noise. Most research has focused on improving the control effect for a feedforward ANC system. However, this paper emphasizes a feedback ANC system based on a signal microphone sensor. There are two main contributions in this study to improve automotive cabin sound comfort. One is that the algorithm of the feedback ANC system using a single microphone sensor without a reference noise signal is proposed based on the Filtered-x Least Mean Square method. The other is that the algorithm applies additive random noise online to estimate the secondary path model. A simulation was implemented based on measured real road noise data, and the simulation results indicate that the proposed feedback ANC system with the single microphone sensor can effectively attenuate road noise. This study shows the feasibility of applying a feedback ANC system in automobiles to increase the cabin sound quality.

## 1. Introduction

Excessive noise can significantly disrupt individuals’ daily routines and work tasks, hampering their ability to effectively communicate and concentrate, consequently diminishing productivity [1]. In more severe instances, prolonged exposure to such noise can lead to adverse health effects. Various sound pressure levels of noise can exert distinct impacts on individuals, affecting their wellbeing differently [2,3]. In everyday life, noise pollution manifests in diverse ways, with persistent exposure to low-frequency noise potentially inducing feelings of irritation, restlessness, and ultimately, detrimental effects on both physical and mental health [4]. Automotive interior noise can cause driver fatigue and induce traffic accidents. Additionally, to a certain extent, it can also destroy the sound quality of automotive music and reduce driving comfort [5,6,7]. In recent years, lightweight design has become a development trend in the automotive industry, as automotive design has increasingly focused on high efficiency and energy saving. The application of lightweight structures in automobiles increases the structural vibration, which inevitably increases the low-frequency noise for drivers and passengers. At the same time, components of tire–road noise mainly focus on the low-frequency range [7]. As the quality of life improves, consumers increasingly demand enhanced comfort features in automobiles, particularly concerning the sound quality within the driver’s cabin [8]. As a result, to mitigate the harmful effects of noise on drivers and passengers, there is a growing emphasis on noise reduction measures [9,10].

Generally speaking, noise control encompasses two main approaches: passive noise control (PNC) and active noise control (ANC) [11,12,13]. PNC technology represents the traditional method of noise reduction, which primarily employs three strategies. Firstly, efforts are made to enhance the automotive structure and manufacturing processes to minimize noise generation at its source. Secondly, noise propagation is managed by installing sound insulation and absorption materials within the automobile. However, this approach is often hindered by challenges such as increased bulkiness, high cost, and degraded performance in attenuating low-frequency noise [14]. Thirdly, noise management can occur at the point of reception, such as through the use of earmuffs in engineering mechanics and helicopter cockpits to mitigate the impact of loud noise on ears. The first and second approaches are commonly employed during the design phase. PNC exhibits a certain efficacy in managing noise across a broad spectrum of high frequencies. However, it is characterized by high cost, bulkiness, and increased weight, which can significantly increase the automotive overall mass. Conversely, due to the longer wavelengths of low-frequency noise and limitations in material thickness, PNC’s effectiveness in mitigating engine and tire–road noise at lower frequencies is constrained. Overall, while PNC demonstrates notable noise reduction capabilities in the medium and high-frequency ranges, its ability to control low-frequency noise is limited.

To overcome the aforementioned challenges, ANC has been put forward to reduce low-frequency noise in various environments, including automotive cabins, aircraft cabins, and industrial settings, to enhance comfort and reduce noise-related fatigue [15,16,17,18]. Research and experimental findings indicate the widespread application of ANC in attenuating noise stemming from engines, powertrains, and tire–road contact [5,7,10]. In 1936, German physicist Paul Lueg pioneered the concept of active noise control by applying for a patent on pipeline active feedforward noise cancellation, marking the inception of ANC [19]. Subsequently, starting in 1953, Olson published a series of seminal papers on active noise control, offering comprehensive insights into the concept and laying a crucial foundation for the technology’s development [20,21]. ANC technology harnesses the principles of destructive interference in acoustics. The essential concept involves eliminating noise by superimposing a calculated reverse soundwave onto the original noise, effectively creating a secondary noise signal with identical frequency, opposing phase, and comparable amplitude. This process introduces an additional secondary sound source, enabling the cancellation of the original noise signal as the two waves intersect and nullify each other. As the actual sound source and environmental conditions vary constantly, noise parameters such as frequency, amplitude, and phase exhibit instability, necessitating continuous adjustment of the parameters within ANC systems to accommodate these changes. Adaptive filters serve this purpose adeptly by autonomously adapting their parameters to minimize the discrepancy between the desired and actual signals. These adaptive filters can be realized through finite impulse response (FIR) filters, infinite impulse response (IIR) filters, and lattice filters. The FIR filter using the least mean square (LMS) algorithm is most commonly implemented for an ANC system.

The ANC system is constructed upon two foundational control methods: feedforward and feedback. In the feedforward ANC, a coherent reference noise input is detected before it reaches the secondary source, while in feedback ANC, the active noise controller endeavors to counteract noise by utilizing an error noise sensor [11]. On the one hand, the feedforward ANC generally exhibits greater robustness compared to the feedback ANC due to its capacity to isolate the reference input from the secondary anti-noise source. As a result, numerous research efforts have concentrated on the development, modification, enhancement, and improvement of the feedforward ANC [22,23,24,25]. On the other hand, the feedback ANC system operates independently of a priori information gathered by the reference microphone sensor, with the attenuated noise level solely dependent upon the error microphone sensor, which offers a notably reduced implementation cost [26]. The more important point is that this approach is immune to multiple noise sources [27], rendering it particularly well-suited for ANC applications within automotive cabins. However, it also has equally apparent drawbacks. The primary drawback lies in its stability problem, similar to that encountered with infinite impulse response (IIR) filters. Additionally, the waterbed effect poses a second weakness, suggesting the theoretical impossibility of simultaneously suppressing noise across all frequencies [28].

In fact, there is not much research on feedback ANC. Wu L. introduced a simplified adaptive feedback ANC system, which directly uses the error signal as the reference in an adaptive feedforward control setup, aiming to mitigate the computational load and programming requirements. The investigations into its convergence properties, advantages, and effectiveness were conducted through simulations and experiments [26]. Luo L. discussed the limitations of conventional ANC methods for nonlinear and broadband noise. He proposed a new approach using the wavelet packet FXLMS (WPFXLMS) algorithm to decompose and independently control predictable parts of broadband noise, demonstrating superior noise cancellation performance through simulations [27]. Wu L. developed an adaptive algorithm designed to eliminate noise amplification in feedback systems by substituting the scalar leaky factor with a real symmetric Toeplitz matrix. This offered improved frequency band adjustment compared to traditional FxLMS and leaky FxLMS algorithms, validated through simulations in an ANC headphone application [28]. Schumacher T. introduced a novel approach to broadband feedback ANC, combining classical non-adaptive and adaptive techniques to attenuate different aspects of ambient noise. This approach offered higher overall noise attenuation performance compared to purely classical or purely adaptive systems. Additionally, a mixed analog–digital implementation was proposed, suitable for low-cost headset devices, while addressing practical hardware constraints [29]. Miyazaki N. explored enhancing a head-mounted ANC system by implementing a virtual sensing technique to achieve greater noise reduction beyond the limitations of error microphone placement. Through experiments and subjective assessment, it was demonstrated that the proposed system achieved higher noise reduction, both objectively and subjectively, compared to conventional systems [30]. Roy T.K. outlined the FxLMS and feedback active noise control algorithms, presenting a novel structure for enhancing noise reduction, with simulation results confirming the effectiveness of the proposed algorithm [31].

The above research focused on theoretical simulation and the application of headphones, while there are few works emphasizing the application of feedback ANC system in an automotive cabin, which is the research objective of this paper. The work presented in this paper aims to develop a feedback ANC algorithm capable of attenuating the low-frequency noise from multiple tire–road noise sources, thereby achieving low interior noise and improving cabin sound comfort. The contributions of this paper encompass two aspects. One aspect involves the utilization of a single microphone sensor to construct a feedback ANC system, while the other involves the online establishment of a secondary path model using additive random noise. It is particularly noteworthy to emphasize the novelty of this research. By employing only one microphone sensor to build a feedback active noise control system, a new approach to system design is introduced, which opens up new possibilities in the field of noise control in an automotive cabin and saves costs for the entire ANC system. Furthermore, by utilizing additive random noise to establish the secondary path model online, not only is the adaptability and robustness of the ANC system enhanced, but also new solutions for real-time noise control in dynamic automotive environments are provided. The remaining sections of the paper are arranged as follows. Section 2 describes the structures and principles of the two standard types of ANC systems. Then, the feedback ANC based on a single microphone sensor is mathematically analyzed in Section 3. The proposed feedback ANC algorithm is presented in Section 4. Simulation results and the discussion are presented in Section 5. Finally, the conclusion is drawn in Section 6.

## 2. Description of an ANC System

**Figure 1:**
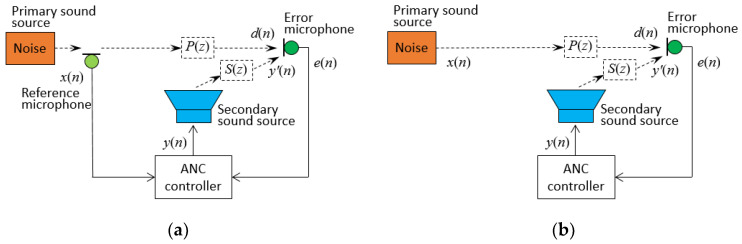
Schematic diagrams of ANC. (**a**) Broad-band feedforward ANC system; (**b**) single-channel feedback ANC system.

ANC systems can be primarily categorized into three types based on the presence of a reference signal: feedforward control system, feedback control system, and hybrid control system [11,12]. In a feedforward control system, a reference sensor and an error sensor are located in different positions, whereas a feedback control system utilizes a single sensor serving as both a reference and error sensor, as depicted in Figure 1a,b, respectively.

In the feedforward ANC system, as illustrated in Figure 1a, the reference signal *x*(*n*) is measured by a reference microphone, and the error signal *e*(*n*) is measured by an error microphone. The two signals jointly drive the ANC controller to generate *y*(*n*), according to a certain algorithm, and the secondary sound source is generated by a loudspeaker after being amplified, canceling the primary sound source at the error sensor. There are two main problems in this system: first, the echo interference problem; second, the delay problem. The echo interference problem refers to the secondary sound waves generated by the loudspeaker propagating in the direction of the reference microphone, which interferes with the accuracy of the reference signal. The delay problem means that there is a delay time for the primary sound source to propagate from the reference sensor to the error sensor, there is a delay time for the secondary sound source to propagate from the loudspeaker to the error sensor, and there is a delay time for the secondary sound source to be generated from the electrical signal to the sound signal. These delay factors are important factors affecting the noise reduction performance of the system. In particular, if the electrical signal delay is greater than the sound propagation delay, the performance of the noise control system will be greatly degraded.

There are two main methods to solve echo interference. The first is to compensate for and neutralize the echo through artificial feedback. The Filtered-X algorithm, known as FxLMS, employs the LMS algorithm to adjust the weight parameters of an adaptive filter. It has garnered significant attention from scholars, due to its computational simplicity and ease of implementation, and it has been greatly researched, developed, and extended. The second method is to utilize adaptive IIR filters. One can use an IIR filter instead of a FIR filter and add an equal number of poles and zeros to the original FIR filter simultaneously, so that the equivalent order of the IIR filter remains unchanged from that of the FIR filter, and the gain is adjusted to make the IIR filter equivalent to the original FIR filter. Thus, the pole of the IIR filter compensates the zero of the echo, and the new zero of the IIR filter cancels the pole of the echo interference.

**Figure 2:**
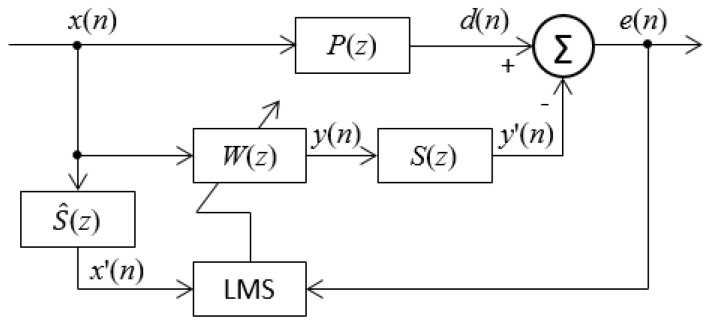
Block diagram of feedforward ANC systems using the FxLMS algorithm.

Figure 2 indicates the block diagram of a feedforward ANC system using the typical Filtered-X LMS algorithm. *X*(*n*) is the noise signal at the *n*th sampling period, which is also used as the reference signal of the FxLMS algorithm; *P*(*z*) is the pulse transfer function in the primary sound propagation path from the noise source to the error microphone sensor; *S*(*z*) is the pulse transfer function in the secondary sound (canceling sound) propagation path from the noise control source (loudspeaker) and the error microphone sensor; $\hat{S}(z)$ is the result of adaptive identification of *S*(*z*); *d*(*n*) and *y*′(*n*) are corresponding responses of the noise source and anti-noise control source at the error microphone sensor, respectively; *d*(*n*) is called the uncontrolled response signal, while *e*(*n*) = *d*(*n*) − *y*′(*n*) is called the residual error noise, namely, the controlled response signal; ***W*** is a weight vector of the adaptive filter. Accurately identifying the acoustic path and transducer is an essential prerequisite for deploying an ANC system through traditional methods. The design of the ANC system filter relies on the transfer function of the acoustic path, ensuring the generation of an effective anti-noise signal.

## 3. Feedback ANC Based on a Single Microphone Sensor

**Figure 3:**
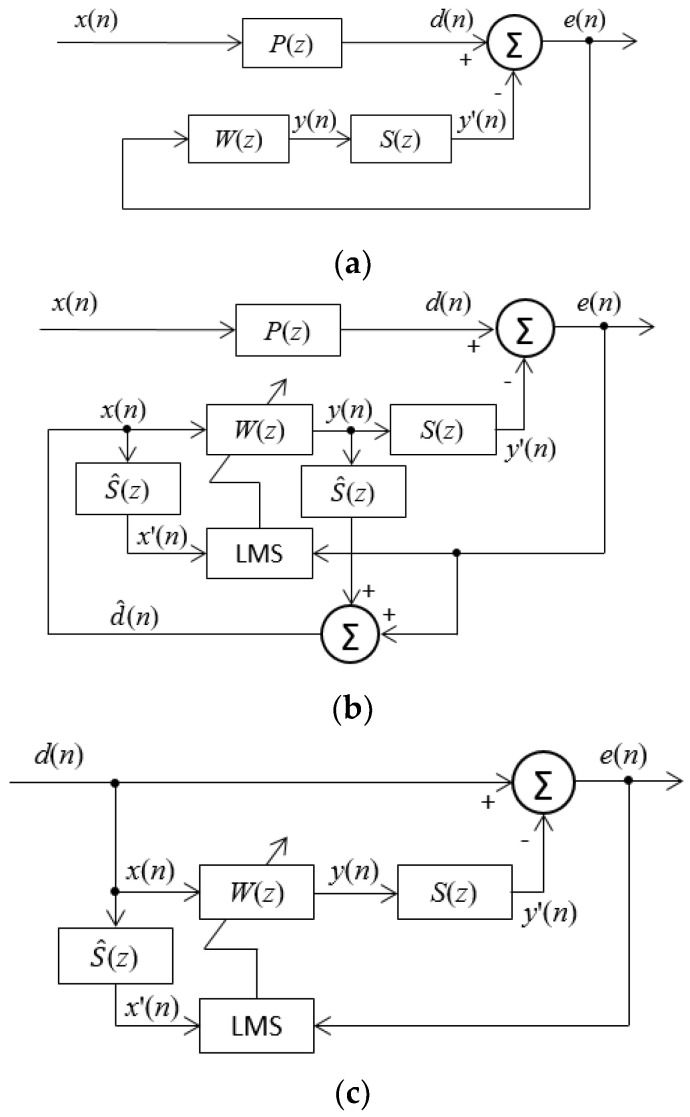
Block diagrams of feedback ANC system using single microphone sensor. (**a**) Equivalent block diagram of Figure 1b. (**b**) Complete block diagram of adaptive feedback ANC system based on the FxLMS algorithm. (**c**) Block diagram of adaptive feedback ANC system for $\hat{S}\left(z\right)=S(z)$.

If the feedforward ANC is applied in automotive settings, a reference microphone sensor or a non-acoustic sensor (narrowband feedforward ANC) is necessary to obtain the noise signal. However, directly accessing the primary noise signal within the automotive interior chamber is not feasible. Moreover, there exist multiple tire–road noise sources. As a result, the feedback ANC system, shown in Figure 1b, is adopted to attenuate noise from road. Figure 3a displays an equivalent, simple, and basic block diagram of Figure 1b, where *d*(*n*) is the primary noise signal at the error microphone sensor, *e*(*n*) is the residual noise signal using the same microphone sensor, and the secondary anti-noise control signal is indicated as *y*(*n*). Two blocks, *W*(*z*) and *S*(*z*), represent the transfer functions of the controller and the secondary sound path from the canceling loudspeaker to the error microphone sensor, respectively.

In the feedback ANC system, illustrated in Figure 3a, accessing the primary noise signal *d*(*n*) during the process of noise cancellation is not feasible since it is intended to be suppressed by the anti-noise sound signal in the secondary path. The solution to this is to utilize an adaptive feedback ANC to identify the primary noise and employ it as a reference signal *x*(*n*) for the adaptive filter, *W*(*z*). Thus, the residual signal after adding anti-noise control signal is expressed in the *z*-domain as below.

(1) $$E\left(z\right)=D\left(z\right)-S\left(z\right)W\left(z\right)E\left(z\right)=D(z)-S(z)Y(z)$$

The above equation can be rewritten as

(2) $$D\left(z\right)=E\left(z\right)+S(z)Y(z),$$

where *E*(*z*) is obtained from the error microphone sensor, *Y*(*z*) is the control signal generated by the adaptive filter in the secondary path, and both are available in the feedback ANC system. To obtain the actual expression of the transfer function *S*(*z*) is crucial to the entire control system. If it is also measurable and approximated by $\hat{S}(z)$, specifically, $\hat{S}(z)\approx S(z)$, the estimated primary noise $\hat{d}(z)$ is considered as a synthesized reference signal *x*(*n*) as follows.

(3) $$X(z)\equiv \hat{D}(z)=E\left(z\right)+\hat{S}(z)Y(z)$$

The block diagram shown in Figure 3b exactly describes the above equation, where the output control sound signal is filtered via the estimation of the secondary path, $\hat{S}(z)$, and then added to the residual signal *e*(*n*) to regenerate the primary noise signal $\hat{d}(n)$. The following equations describe the detailed calculation process of *x*(*n*) and *y*(*n*).

(4) $$x\left(n\right)\equiv \hat{d}\left(n\right)=e\left(n\right)+\sum_{m=0}^{M-1}{\hat{s}}_{m}y\left(n-m\right),$$

(5) $$y\left(n\right)=\sum_{l=0}^{L-1}w_{l}(n)x\left(n-l\right),$$

where ${\hat{s}}_{m},m=0,1,\ldots ,M-1$ are the coefficients of the *M*th order FIR filter $\hat{S}(z)$ used to estimate the secondary path, which can be determined either offline or online [32], $w_{l}(n),l=0,1,\ldots ,L-1$ are the coefficients of the adaptive FIR filter *W*(*z*) at time *n*, and *L* is the order of *W*(*z*). These coefficients are updated by the FxLMS algorithm as

(6) $$w_{l}\left(n+1\right)=w_{l}\left(n\right)+\mu x^{\prime }(n-l)e\left(n\right),\;l=0,\;1,\;\ldots ,\;L-1,$$

where *μ* is the step size, and the filtered reference signal *x*′(*n*) is

(7) $$x^{\prime }\left(n\right)=\sum_{m=0}^{M-1}{\hat{s}}_{m}x\left(n-m\right).$$

Through the above analysis, if the transfer function of the secondary path *S*(*z*) is successfully obtained by $\hat{S}(z)$, namely, $\hat{S}\left(z\right)=S(z)$, then *x*(*n*) = *d*(*n*) can be determined, and then the adaptive feedback ANC system shown in Figure 3b can be transformed into Figure 3c, which has the same form of an adaptive feedforward ANC system.

## 4. Feedback ANC with Online Secondary Path Modeling

**Figure 4:**
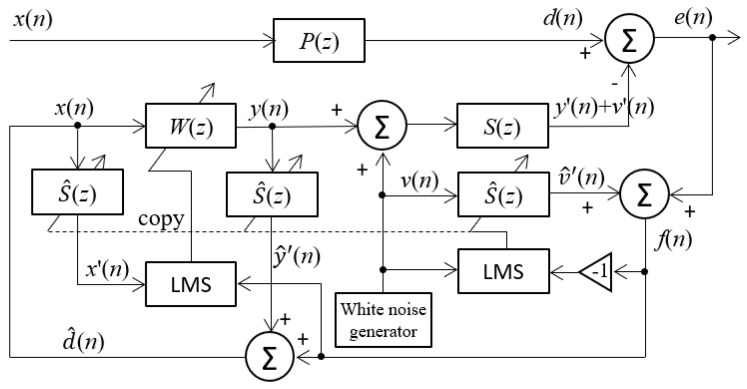
Block diagram of feedback ANC system including online secondary path modeling via adopting additive random noise.

The proposed approach in the paper is based on the feedback ANC system, as shown in Figure 1b, where the primary noise signal *d*(*n*) is not accessible because there is no reference microphone sensor to measure it. One of the main challenges for improving automotive cabin sound comfort with the feedback ANC system is to restore the primary noise signal, *d*(*n*), by using the residual error noise signal, *e*(*n*), measured by the single error microphone sensor. Because the secondary path from the active sound source (the loudspeaker) to the error microphone is usually nonlinear and time-varying due to the driving state and surroundings, another challenge is how to deal with this change, model the secondary path, and guarantee the convergence stability and precision of the feedback ANC system. Compared to offline modeling before implementation of the ANC algorithm, adopting online secondary path modeling is a good method to meet these requirements. One of the popular online secondary path modeling techniques, the use of an additive random noise generator, was presented by Eriksson and Allie [33]. This technique involves using a random noise generator to produce a white noise *v*(*n*) with a zero mean that remains uncorrelated with the primary noise. Subsequently, this internally generated noise signal is combined with the secondary control signal *y*(*n*), generated by the adaptive ANC filter *W*(*z*), to activate the secondary source. The proposed approach to improving automotive cabin sound comfort is to employ the additive random noise technique into the feedback ANC system using a single microphone sensor, illustrated in Figure 4.

Comparing the block diagram in Figure 4 with Figure 3c, the online secondary path modeling with additive random noise is connected with the secondary control path and the error signal path. The adaptive filter $\hat{S}(z)$ is connected in parallel with the secondary path to model *S*(*z*), and there are another two $\hat{S}(z)$ filters on the left side in the block diagram, which are used to produce inaccessible primary noise signal *d*(*n*), similar to the structure of Figure 3c. The input signal used to establish $\hat{S}(z)$ is solely the random noise *v*(*n*), instead of the combination of the secondary signal *y*(*n*) + *v*(*n*).

Thus, the residual error is expressed as

(8) $$e\left(n\right)=d\left(n\right)-y^{\prime }\left(n\right)-v^{\prime }\left(n\right)=d\left(n\right)-s\left(n\right)*y\left(n\right)-s\left(n\right)*v\left(n\right),$$

where

(9) $$y^{\prime }\left(n\right)\equiv s\left(n\right)*y\left(n\right)$$

is the control signal in the secondary path for attenuating the raw noise, and

(10) $$v^{\prime }\left(n\right)\equiv s\left(n\right)*v\left(n\right)$$

is the signal component of the additive random noise used for path modeling. At the same time an estimation $v^{\prime }\left(n\right)$ of Equation (10) by means of the estimated model of the secondary path $\hat{S}(z)$ is

(11) $${\hat{v}}^{\prime }\left(n\right)=\hat{s}\left(n\right)*v\left(n\right),$$

where $\hat{s}\left(n\right)$ is the impulse response of the filter $\hat{S}(z)$ of the secondary path.

According to the connection in Figure 4, the residual error for the modeling filter $\hat{S}(z)$ is calculated as

(12) $$f\left(n\right)=e\left(n\right)+{\hat{v}}^{\prime }\left(n\right)\;=d\left(n\right)-y^{\prime }\left(n\right)-v^{\prime }\left(n\right)+{\hat{v}}^{\prime }\left(n\right)\;=(d\left(n\right)-y^{\prime }\left(n\right))-(v^{\prime }\left(n\right)-{\hat{v}}^{\prime }\left(n\right)).$$

In the above equation, the first term in the parentheses is the residual error of the primary path, while the second term is the residual error for online modeling of the secondary path. These two residual errors are not correlative with each other because the primary road noise and the additive random noise are independent. Therefore, the first term in parentheses in *f*(*n*) does not affect the convergence and accuracy of modeling the filter $\hat{S}(z)$ based on the theory of adaptive filter when the LMS algorithm is implemented.

On the other side, since it is impossible to access the primary noise *d*(*n*), the estimated primary noise $\hat{d}(z)$ is reconstructed as a synthesized reference signal *x*(*n*) as expressed in Equation (3), if $\hat{S}(z)\approx S(z)$. As a result, after introducing the additive random noise, Equation (3) can be modified as

(13) $$x\left(n\right)\equiv \hat{d}\left(n\right)=f\left(n\right)+{\hat{y}}^{\prime }\left(n\right)\;=\left(d\left(n\right)-y^{\prime }\left(n\right)\right)-\left(v^{\prime }\left(n\right)-{\hat{v}}^{\prime }\left(n\right)\right)+{\hat{y}}^{\prime }\left(n\right),$$

where

(14) $${\hat{y}}^{\prime }\left(n\right)\equiv \hat{s}\left(n\right)*y\left(n\right).$$

It can be seen from Equation (13) that if $\hat{S}(z)\approx S(z)$, then ${\hat{y}}^{\prime }\left(n\right)\approx y^{\prime }\left(n\right)$, and Equation (13) is simplified as

(15) $$x\left(n\right)\equiv \hat{d}\left(n\right)=f\left(n\right)+{\hat{y}}^{\prime }\left(n\right)\;=d\left(n\right)-\left(v^{\prime }\left(n\right)-{\hat{v}}^{\prime }\left(n\right)\right),$$

in which the synthesized reference signal *x*(*n*) comprises the residual error when estimating the filter model of $\hat{S}(z)$. This leads to the divergence of updating the weight coefficients of the adaptive ANC filter *W*(*z*), which is quite different from that of applying the feedforward ANC.

The paper presents an approach to addressing the above problem. Specifically, the feedback ANC system remains inactive during the process of online secondary path modeling, and it starts to work after model estimation of the secondary path is completed. The duration of constructing the $\hat{S}(z)$ model is short, as demonstrated in Section 4.

## 5. Simulation Study Using Real Road Noise Data and Discussion

In this section, the computational simulation is described, and the results are presented to demonstrate the effectiveness and performance of the proposed feedback ANC algorithm. Unlike most research papers, the primary noise employed in the simulation is not white noise or composite harmonic waves with several different frequencies, which are generated by a computer, but real measured road noise. A passenger car was driven at an almost constant speed of 60 km/h on a rough asphalt city road, and the noise around driver’s ears was measured using a signal microphone sensor with the sampling rate of 8000 Hz.

**Figure 5:**
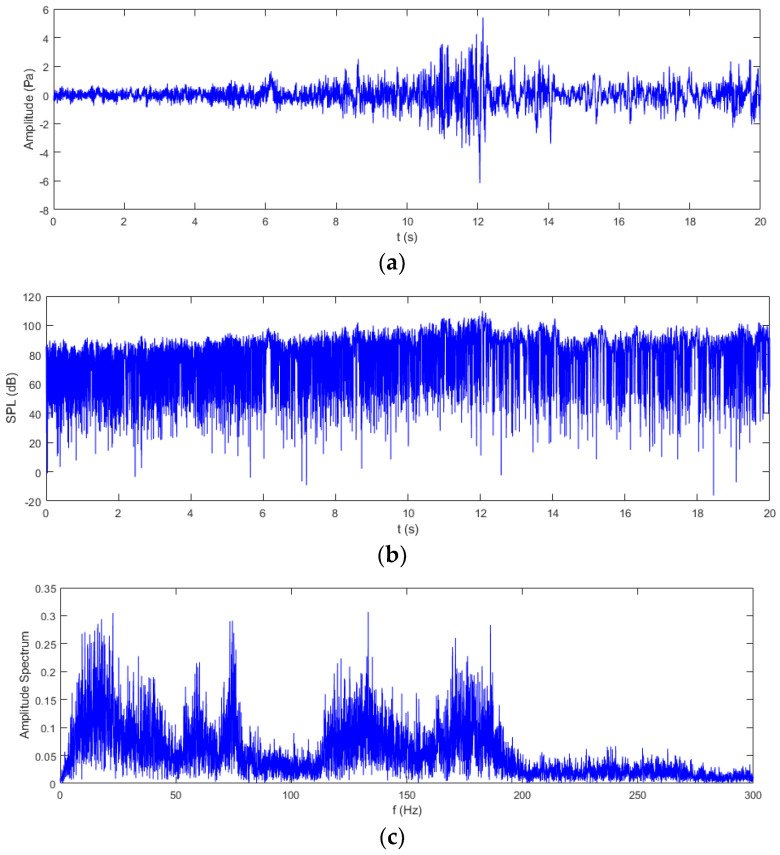
Representations of real road noise data. (**a**) Sound pressure. (**b**) Sound pressure level (SPL). (**c**) Amplitude spectrum in frequency domain.

The real road noise data are represented in Figure 5a. According to the sensitivity of the sensor, the measured voltage signal was converted into sound pressure value, as shown in Figure 5b. It can be seen that the sound pressure of the noise is low in the beginning; then, it gradually becomes larger after 5 s, reaching the maximum value around 12 s. The sound pressure is also expressed by the sound pressure level (SPL), as shown in Figure 5b. The SPL of the noise exceeds 80 dB, reaching even greater than 100 dB around 12 s. Note that the figure shows low SPL values because a moving average window was not applied to the raw noise sound pressure signal. In order to investigate the frequency components of the raw road noise signal, the FFT transform was implemented, and the amplitude spectrum with a frequency range up to 300 Hz is illustrated in Figure 5c. The plot indicates that the frequencies of the road noise concentrate in the low-frequency range less than 200 Hz, which is due to the interaction between the road and tires while driving [9]. Many frequency components are located in the range less than 50 Hz and around 130 Hz and 175 Hz, respectively, and there are two peaks near 60 Hz and 70 Hz.

**Figure 6:**
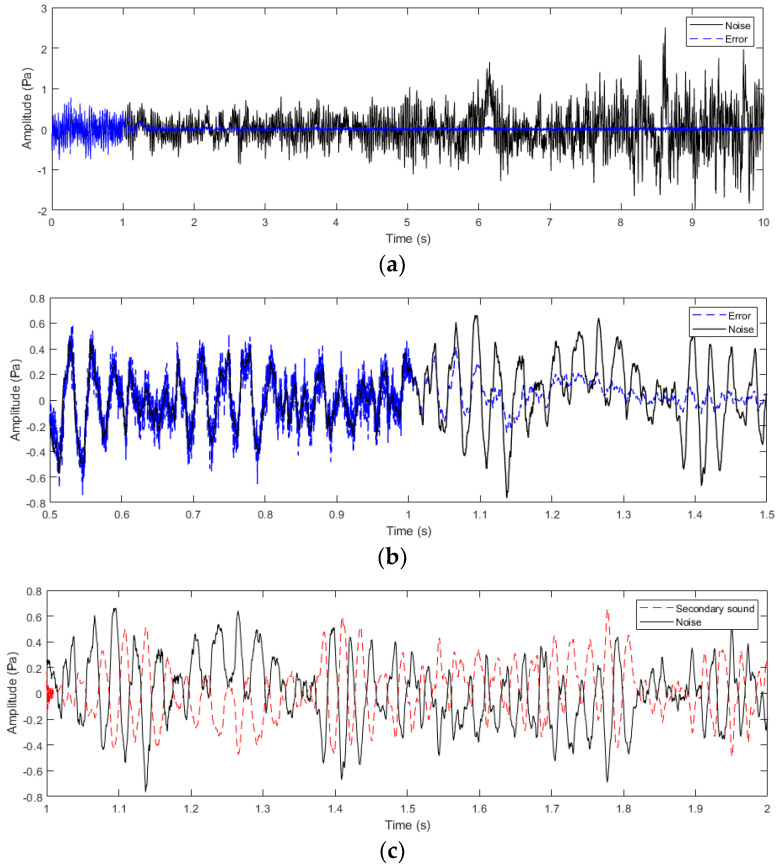
Simulation result of applying feedback ANC for the road noise. (**a**) The road noise and the residual signal during 0 s to 10 s. (**b**) Comparison of the control effect before and after 1 s: waveforms of the road noise and the residual error. (**c**) The road noise and the generated anti-noise sound in the secondary path.

A simulation model was constructed based on the proposed feedback ANC system with online secondary path modeling using additive random noise. The sampling rate is the same as that of the measured raw noise data, 8000 Hz. For example, to attenuate 50 Hz noise, one period takes 0.02 s, and 160 samples can cover an entire period. Accordingly, the length of the adaptive filter *W*(*z*) is set to 200. During simulation, the first stage from 0 s to 1 s involves executing online secondary path modeling to obtain the estimation of S(z), $\hat{S}(z)$, which the feedback ANC system does not carry out. Then, after 1 s, in the second stage, the feedback ANC starts to suppress the road noise by using the weight coefficients of $\hat{S}(z)$ estimated in the first stage. Figure 6a depicts the simulation results from 0 s to 10 s. It can be seen clearly that the residual error overlaps the original road noise signal in the first 1 s due to no noise attenuation effect, and the raw noise can be effectively suppressed after 1 s when feedback ANC is applied. In order to investigate the transient process of two stages in more detail, waveforms of the measured original road noise and the residual error are represented during the period between 0.5 s and 1.5 s, as shown in Figure 6b. The residual error signal before 1 s is a combination of the original road noise and the additive random noise, while after 1 s, it is a superposition of the former and the active noise control signal. With time passing, the active noise control signal becomes more effective, resulting in the residual error signal becoming weaker and weaker. Figure 6c demonstrates the waveform of the generated anti-noise signal at the error microphone sensor, which has an approximately opposite sign to the raw road noise with convergence of weight coefficients of the adaptive filter *W*(*z*). Note that the initial anti-noise signal after 1 s in Figure 6c contains high frequency components due to the existence of the previous additive random noise signal.

**Figure 7:**
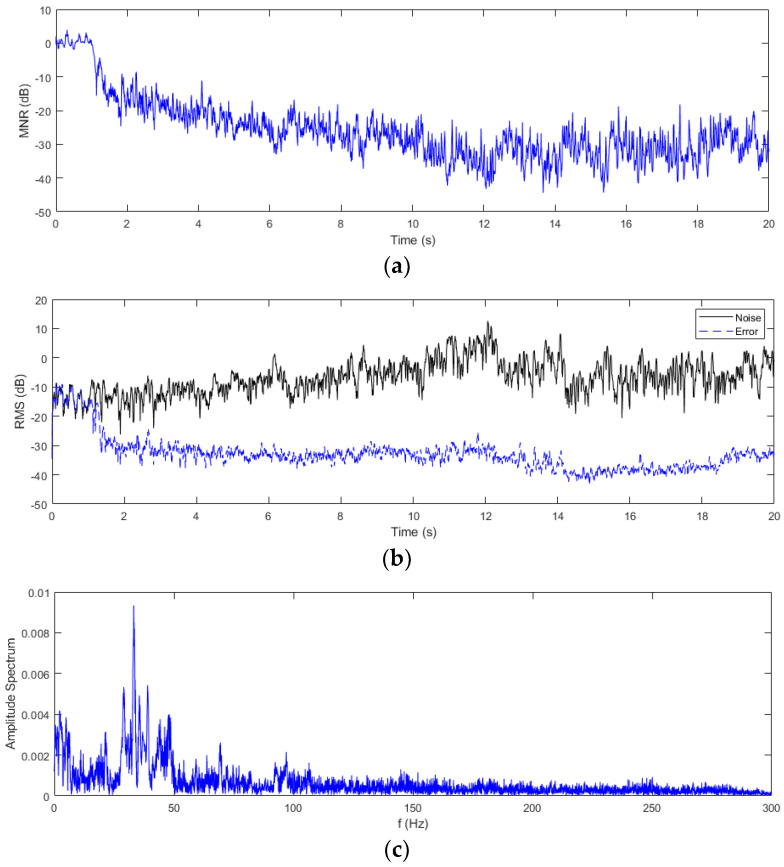
Evaluation of the simulation result of the proposed feedback ANC system. (**a**) Evaluation of the control effect by applying feedback ANC for the road noise. (**b**) Comparison of the RMS values of the road noise and the residual error. (**c**) Amplitude spectrum of the residual error in the frequency domain.

In order to evaluate the effectiveness of noise attenuation by using the proposed feedback ANC system, the mean noise reduction (MNR) is defined as the ratio of the average residual error power to the average road noise power [34,35,36], as shown in Equation (16). The MNR curve is shown in Figure 7a, where the MNR value remains almost above 0 dB in the first 1 s, since there is no noise control, and the additive random noise exists. However, after 1 s, the MNR value steeply drops below −10 dB due to the effectiveness of the feedback ANC, and it fluctuates around −30 dB after 10 s.

(16) $$MNR\equiv 10{log}_{10}\left(\frac{E[e^{2}(n)]}{E[d^{2}\left(n\right)]}\right)$$

On the other hand, the root mean square (RMS) values of the original road noise and the residual error are compared as shown in Figure 7b. Similarly, the RMS value of the latter was reduced by than more 30 dB compared to that of the former. From the viewpoint of frequency domain analysis, the frequency components of the residual error signal during 1 s to 20 s are illustrated in Figure 7c. Compared with the frequency components of the original road noise signal shown in Figure 5c, the noise components above 50 Hz are noticeably attenuated, except for a small peak around 33 Hz.

Based on the above simulation result, some discussion is given here. Firstly, through analysis of the measured real road noise data, it is evident that there is a significant noise level in the passenger car cabin during normal driving speed, with the noise SPL exceeding 80 dB and sometimes even reaching above 100 dB. The FFT analysis reveals that the road noise is primarily concentrated on frequencies below 200 Hz, attributed to interactions between the road and tires during driving. It is very difficult for the traditional PNC to attenuate such low frequency noise. Thus, it reasonable to apply the feedback ANC to the automotive cabin. Secondly, the proposed feedback ANC system is implemented through a two-stage process. During the period from 0 to 1 s, the algorithm online estimates the model of the secondary path $\hat{S}(z)$, while the ANC system remains inactive. Activating it during this period would cause the divergence of the FxLMS algorithm, since the virtual reference signal *x*(*n*) contains the additive random noise, as expressed in Equation (15). Once the adaptive filter $\hat{S}(z)$ is constructed in a short moment, the feedback ANC system initiates noise reduction on the measured real road noise signal. Thirdly, the simulation result, illustrated in Figure 6, depicts that, in the stage of online secondary path modeling, the additive random noise coincides with the measured low frequency noise signal. Subsequently, in the second stage, effective noise suppression is achieved, with convergence of the ANC adaptive filter weight generation and a gradual reduction in the residual error over time. Evaluation indices such as the mean noise reduction and root mean square value are adopted to assess the effectiveness of the proposed algorithm. The results indicate that, over time, both evaluation indices decrease by 30 dB and 15 dB, respectively, which validates the efficacy of the algorithm. It also can be seen that the simulation result in the paper is superior to the noise attenuation of up to 20 dB for low frequencies in Schumacher’s research [29] and to the noise reduction performance of about 20 dB in Miyazaki’s research [30], both of which also adopted a feedback ANC system. In the case of using the popular feedforward ANC system, Kang obtained 4 to 14 dB of booming noise reduction in an experiment [6], Pi’s real automotive experiments showed that a maximum noise reduction of 9 dB was reached in the frequency range of 70–120 Hz [10], and Tang’s simulation result showed that an MNR value of less than 25 dB could be obtained [34]. As a result, the simulation result in this work is also superior to those applying a feedforward ANC system in simulations or experiments.

## 6. Conclusions

Following the analysis of traditional feedforward and feedback ANC systems, this paper introduces a novel feedback ANC algorithm, which incorporates online secondary path modeling, utilizing a single microphone sensor to enhance automotive cabin sound comfort. The algorithm employs additive random noise in real time to estimate the secondary path model, even in the presence of original road noise. Following this, the ANC adaptive filter is updated using the FxLMS algorithm, while simultaneously generating anti-noise to cancel out the road noise. The simulation results underscore the efficacy of the proposed feedback ANC algorithm, which utilizes a single microphone sensor, in substantially attenuating road noise by an impressive 30 decibels.

Feedback ANC systems offer effective noise reduction by using a microphone sensor to detect residual error noise and adjusting the anti-noise signal accordingly. However, they also come with several limitations, including instability, limited frequency range, latency, and trade-offs. Feedback ANC systems can be prone to instability, particularly when dealing with complex or rapidly changing noise environments. Instability can lead to undesirable oscillations or even system failure. This compromises performance and reliability. Feedback ANC systems may struggle to effectively attenuate noise across a wide frequency range. They are typically more effective at reducing noise at lower frequencies. However, their performance may be limited at higher frequencies. While suitable for road noise reduction, they may not be as effective for reducing harshness. Feedback ANC systems introduce a delay between the detection of the residual error noise and the generation of the anti-noise signal. This latency can limit the system’s ability to respond quickly to changes in the noise environment, especially in dynamic situations. Addressing this challenge remains important for road noise reduction. There are often trade-offs between noise reduction performance, system stability, and implementation complexity in feedback ANC systems. Achieving optimal performance may require careful balancing of these factors. Compromises may be necessary in some areas to achieve the desired results.

Due to the limitations mentioned above, there exists a challenge to implement the proposed algorithm in a corresponding experiment and in a real automobile. Future work can be divided two steps. The first step involves conducting the algorithm experiments in laboratory surroundings, where real measured noise is simulated using loudspeakers, while another set of speakers serves as the source for generating anti-noise signals, providing a controlled environment for algorithm testing and refinement. In feedback ANC experiments, research efforts should be directed towards improving system stability, reducing latency, real-time implementation, and optimizing the trade-off between noise reduction performance and system complexity. The second step is to execute the algorithm in a real automobile to validate the effectiveness. One of the key challenges in the in-car ANC system is how to deal with the dynamic and unpredictable various noise sources, including not only tire–road noise but also engine noise and wind noise. The ANC system must be able to adapt to these changing noise conditions in real time to provide effective noise reduction to improve cabin sound comfort.

## Data Availability

Data are contained within the article.
